# *Care Coordination: Empowering Families*, a Promising Practice to Facilitate Medical Home Use Among Children and Youth with Special Health Care Needs

**DOI:** 10.1007/s10995-018-2477-2

**Published:** 2018-02-14

**Authors:** Lisa Gorman Ufer, Julie A. Moore, Kristen Hawkins, Gina Gembel, David N. Entwistle, David Hoffman

**Affiliations:** 1 Michigan Public Health Institute, 2501 Jolly Rd. Suite 180, Okemos, MI 48864 USA; 2 Region 4 Midwest Genetics Collaborative, Okemos, MI USA; 3 Department of Psychology, Malone University, Canton, USA

**Keywords:** Care coordination, Medical home, Training, Families, CYSHCN

## Abstract

*Introduction* This paper describes the care coordination training program and results of an evaluation from its pilot in seven states. Despite the importance of practice-based care coordination, only 42.3% of children with special health care needs (CYSHCN) met all needed components of care coordination as defined by the Maternal Child Health Bureau. Recognizing that children with medically complex conditions often have lower rates of achieving care coordination within a medical home, the Region 4 Midwest Genetics Collaborative worked with families to develop a training to empower families in care coordination. The *Care Coordination: Empowering Families*(CCEF) training provides families with the knowledge, tools, and resources to engage with health, education and family support systems. This article gives an overview of the training and comprehensive evaluation. *Methods* Participants were family caregivers of children with genetic conditions and other special health care needs recruited in one of seven pilot states. Evaluation data were collected from 190 participants prior to and immediately following the training. An additional follow-up assessment one full year post training was completed by 80 participants (a response rate of 42%). *Results* Families who attended the training report being the primary source of care coordination for their children and 83.7% see their role in their child’s healthcare changing as a result of the training. The findings suggest that peer support and communication with providers increased as a result of the training over the course of the study. The data suggest that the training impacted how the family interacts with the child’s doctor, including initiating conversations to prepare their child for transition to adult health care. Further, families report system-level improvements 1 year later compared to the pre-training assessment. *Discussion* CCEF training is a promising practice for facilitating medical home use among CYSHCN.

## Significance

Effective care coordination has been associated with positive outcomes for families and children, and it is generally expected that healthcare professionals will provide care coordination services as part of the family-centered medical home. This paper describes a training designed to empower parents as the main coordinator of their child’s care in the event that the care providers do not or cannot offer coordination support. The comprehensive evaluation of the training shows that parents can bring about positive change when they have the knowledge, skills, and resources for interacting with the healthcare system.

## Introduction

Children or youth with special health care needs (CYSHCN) are described by the Maternal and Child Health Bureau (MCHB) as “those who have or are at increased risk for a chronic physical, developmental, behavioral, or emotional condition and who also require health and related services of a type or amount beyond that required by children generally” (McPerson et al. 1998). These families often manage multiple systems of care (e.g., primary, specialists, therapies, education) without a central entry point (AAP 2005). A Medline review of publications found that patients with five or more chronic conditions may encounter up to 14 physicians in one calendar year (Vogeli et al. 2007). Given this complicated landscape, effective care coordination across care providers is paramount for families of CYSHCN to maintain their children’s optimal health by avoiding fragmented or duplicated healthcare services. For families with children with complex conditions (e.g., genetic conditions identified through newborn screening), both the need for, and burden of, care coordination is even greater (Cooley et al. 2013; Kuo et al. 2011; Golden and Nageswaran 2012).

This paper describes a care coordination training that empowers families and results of an evaluation from its pilot in seven states. Effective care coordination, which facilitates the linkage of children and their families with appropriate services and resources to achieve good health (COCWD 2014), has been associated with positive outcomes for families and children (Lawson et al. 2011; Farmer et al. 2011; Turchi et al. 2009; Miller et al. 2013). In a survey of 780 patients, care coordination was endorsed as one of the most important elements of the family-centered medical home (Wexler et al. 2012).

Despite the broad consensus around the importance of practice-based care coordination, especially for CYSHCN, many barriers keep practices from offering it (McAllister et al. 2007). In the U.S., only 42.3% of CYSHCN met all components of care coordination as defined by the Maternal Child Health Bureau (NS-CSHCN 2009/10). Children with more medically complex conditions often have lower rates of achieving care coordination within a medical home, as the ideal location of a medical home might shift over time (e.g., between specialists and primary care; Raphael et al. 2013). In the U.S., it is estimated that 43.0% of all CYSHCN have a medical home, compared to only 34.6% of those children with complex needs (National Survey of Children with Special Health Care Needs 2009/10). A lack of effective care coordination may have a profound effect on higher needs patients whose conditions may require long term follow-up care with specialists (Sahai et al. 2010).

### Purpose

This paper describes the *Care Coordination: Empowering Families* (CCEF) training and results of an evaluation from its pilot program in seven states. The program was developed at the request of and with family stakeholders who reported being the only consistent managers of care for their CYSHCN. Effective care coordination is not universally available in medical practices and gaps may exist among medical offices and other services (e.g., education, housing, transportation, respite) that children with complex conditions may need. The AAP Council on Children with Disabilities 2005 policy statement affirms that families of CYSHCN should have the opportunity to lead and/or be proactive participants of their child’s care coordination team. Traditionally, the onus has been on healthcare professionals to provide care coordination services as part of the medical home (e.g., McAllister et al. 2007; Committee on Hospital Care and Institute for Patient-and-Family-Centered Care 2012; Gupta et al. 2004; Moore and Tonniges 2004). While many of these initiatives involve families in phases of planning and implementation, few focus on supporting parents as the main coordinator of their child’s care, a role that families often fill if care providers do not or cannot offer coordination support (Gupta et al. 2004; Berry 2015). To fulfill this role, families need adequate knowledge in their child’s condition, care coordination skills, knowledge of the medical home concept, and access to healthcare resources. The need for additional knowledge and skills was identified as a priority for families in the Region 4 Midwest Genetics Collaborative (Region 4 Midwest) and led to the development of the training, CCEF.

### Theoretical Model

**Fig. 1:**
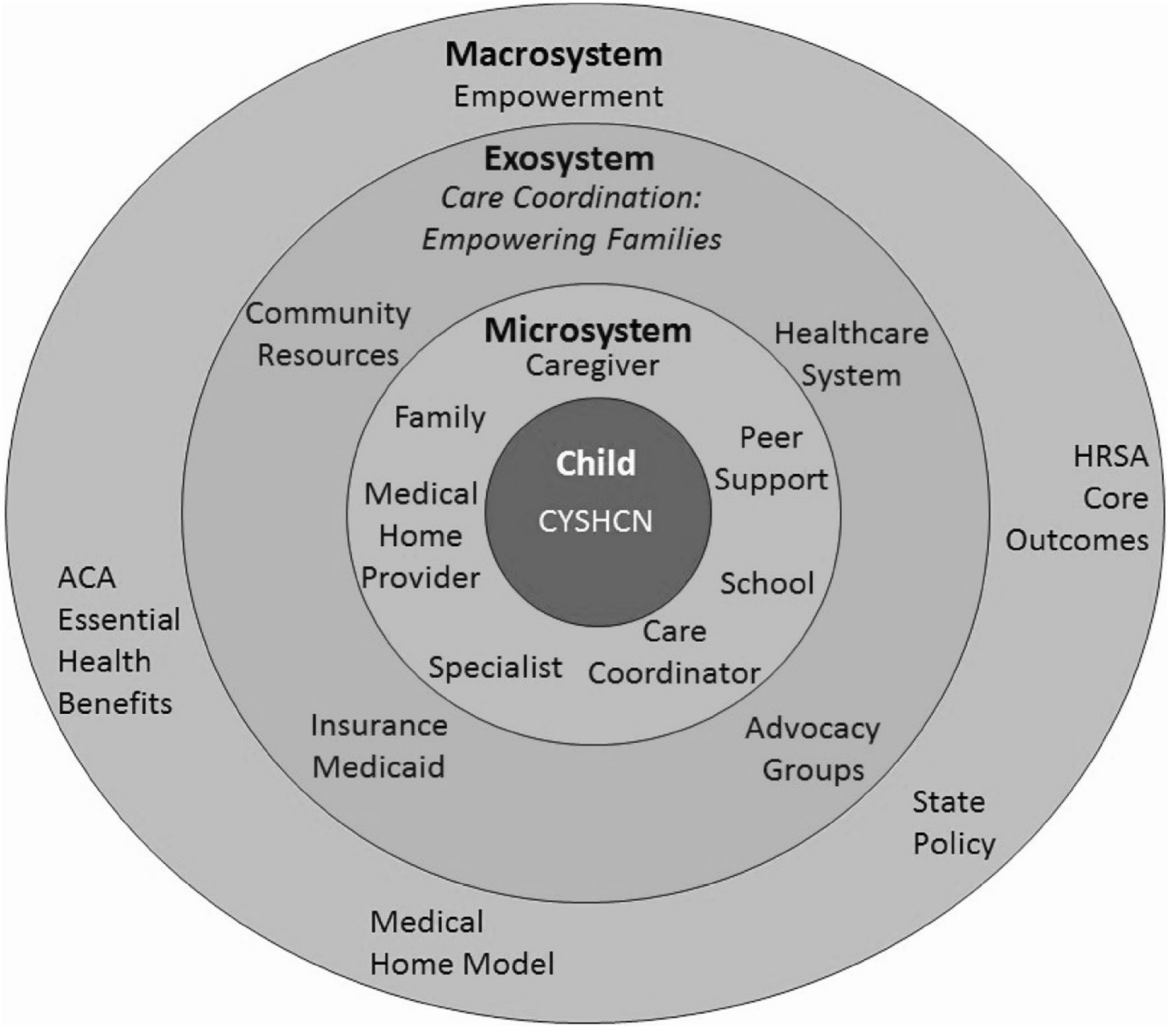
Illustration of CCEF and support in CYSHCN systems with the ecological model

Bioecological theory (Bronfenbrenner 1977; Bronfenbrenner and Morris 2006) was the framework for CCEF curriculum development, operationalization and evaluation. As depicted in Fig. 1, the children and youth with special health care needs (CYSHCN) are seen as part of a dynamic developmental system wherein relations among levels are seen as the basis of development. By taking a systems and family-centered approach to the training, Region 4 Midwest recognized the strength and capabilities of families, promoted greater parent/professional partnership with the healthcare and service delivery systems, and empowered parents as a way to provide help and information in relation to the coordination of care for their children (Dunst et al. 2007). Region 4 Midwest aimed to develop an intervention that would empower families to engage with the healthcare system which is achieved by increasing the parents’ knowledge about medical homes and care coordination through the training, and specifically by empowering them as experts of their children. As a result, Region 4 Midwest expected that the CCEF training curriculum would change the way that families interact with the systems that promote better health.

### Development of a Family-Centered Care Coordination Training

The CCEF training curriculum, implementation plan, recruitment strategies, training evaluation, and sustainability plan were developed in close collaboration with the Family Forum of Region 4 Midwest (a group of parents of children with genetic conditions) and clinical partners from seven states. The curriculum includes activities and opportunities within the training day to develop skills and use tools aimed at increasing parenting self-efficacy (Dunst and Trivette 2009) and equips families with the knowledge and belief that they can influence events that affect the health and development of their children.

### Training Model

The 8 h, interactive training can be provided to up to 25 participants at one time. Participants should be parents of children who have been identified as having either a genetic condition or special health care need. Participants were provided a $150 gift card in recognition of their investment of time and to assist with transportation and childcare costs. Take-home materials include a participant workbook with additional resources, *Partnering with your Doctor: The Medical Home Approach* booklet and a personal journal.

**Fig. 2:**
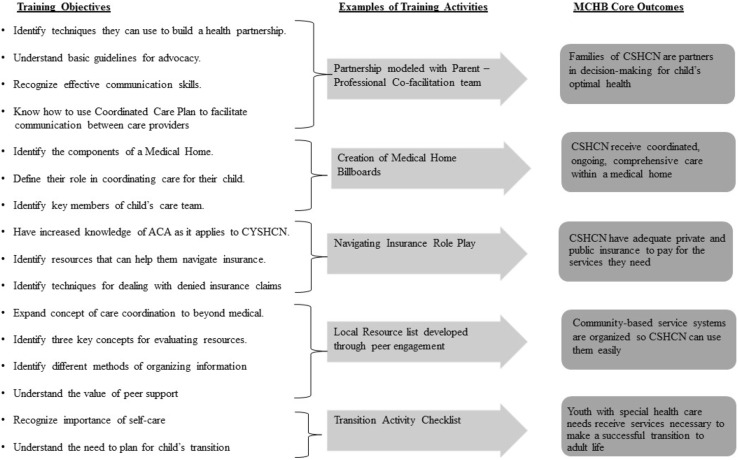
CCEF objectives with examples of training activities as they relate to MCHB core outcomes

The CCEF training curriculum includes eight core training components with 16 learning objectives, each aimed toward accomplishing MCHB core outcomes. Figure 2 illustrates the relationship of training objectives to intended outcomes and provides examples of training activities. The training highlights the significance of family-professional partnerships in both training content and by using a parent/professional facilitation team (at least one facilitator must be a parent of a child with a genetic condition or other special health care need). The importance of peer support for parents of children is emphasized by developing a community of parents with common experiences and needs in the training itself (Shilling et al. 2013 for review). In addition to meeting parents who have a shared social identity, the training provides an opportunity to learn practical information and be inspired by others with similar experiences.

## Methods

### Procedure

Evaluation procedures were approved by the Michigan Public Health Institute Institutional Review Board. Data were collected from training participants at three time points: pre-assessment, post-training (immediately after training), and 1 year follow-up. Participants were asked to create a personal identification code using a series of three questions to link surveys across time without accessing identifiable information. The pre-assessment and 1 year follow-up surveys mirror each other to evaluate long-term training impact. Participants completed both of these surveys online (paper copies were mailed upon request). Study data were collected and managed using Research Electronic Data Capture (REDCap), a secure, web-based application designed to support data capture for research studies (Harris et al. 2009). The intent of the post-training survey was to determine if training objectives were met, assess readiness for change, and to improve the quality of the training. This survey was distributed in paper format immediately after the training. Participation in the training and evaluation of the training was voluntary. Participants were encouraged to complete three surveys to help us improve our training and measure if we accomplished our training goals.

Parent coordinators partnered with families and organizations such as Family to Family Health Information Center, Title V, Sickle Cell Disease Associations, Family Voices, clinics, and hospitals to recruit participants. To be eligible to participate in the training, individuals had to identify themselves at registration as a parent or primary caregiver of a child with a genetic condition. After the initial training funded by the genetics collaborative, partners expanded the inclusion criteria to include parents and caregivers of all CYSHCN.

### Participants

A total of 190 caregivers participated in one of ten CCEF trainings in 2013. All training participants completed the pre and post-training assessments, and 80 participants (42% response rate) completed the 1 year follow-up assessment. Respondents to the follow-up survey did not differ across many demographic variables compared to training participants who did not respond (see Table 1). Further, respondents and non-respondents to the follow-up had similar rates of achieving the MCHB core outcomes at pre-assessment. The participants’ children with special health care needs represented a wide range of genetic and other medical conditions and developmental delays. Distribution of racial groups is comparable to the population in the seven-state region based on U.S. Census Bureau (2010). As a result of recruitment efforts to improve access to genetic services for underserved populations, nearly one in four training participants identified with a minority race or ethnic group. Indeed, White participants are slightly under-represented (76.5% CCEF compared to 82.4% seven-state population) and Black participants over-represented (20.5% CCEF compared to 9.9% seven-state population) in the CCEF participant sample. Participants represented a broad spectrum of income levels.

**Table 1:** Participant demographic characteristics at pre-assessment and 1 year follow-up

Variable	Pre-assessment N = 190	1 Year follow-up N = 80
Child’s age in years		
Mean (SD)	8.9 (5.7)	9.6 (5.3)
Range	0–27.0	1.0–28.0
Average age of diagnosis in years		
Mean (SD)	1.8 (2.5)	1.9 (2.6)
Range	0–12.0	0–12.0
Number of health conditions (check all that apply)		
Mean (SD)	2.4 (1.9)	2.3 (1.8)
Range	0–9	0–8
Number of developmental conditions (check all that apply)		
Mean (SD)	1.4(1.8)	1.3 (1.7)
Range	0–7	0–7
Race and ethnicity (check all that apply)		
White	76.5%	77.1%
Black	20.5%	20.0%
Asian	1.8%	2.9%
American Indian or Alaskan	0.6%	1.4%
Arabic	0.6%	0%
Other	1.0%	0%
Hispanic	6.0%	7.0%
Income		
< $20,000	26.6%	22.5%
$20,000–$30,000	11.7%	8.8%
$30,001–$40,000	14.4%	15.0%
$40,001–$50,000	10.6%	8.8%
> $50,000	36.7%	45.0%
Insurance (check all that apply)		
Medicaid	65.8%	61.3%
Employer/union	47.9%	53.8%
S-CHIP (state)	10.0%	18.8%
Military	0.5%	0%
Uninsured	1.6%	1.3%
Other	6.8%	10.0%
Number of children in home		
Mean (SD)	2.4 (1.4)	2.5 (1.4)
Range^a^	0–10	1–6

^a^Six participants reported 0 children living in the home. These participants were other caregivers, step-parents, or parents of an older child who was currently living outside of the home

### Assessment and Measures

The evaluation tools assessed the training from several perspectives. Table 2 provides an overview of measures and the data collection schedule. First, a post-training assessment was used to understand training quality and provide opportunity for quality improvement efforts in the pilot. The post-training assessment included measures of training satisfaction, content knowledge, and training objectives. Second, training impact was assessed on three levels: readiness for change, care coordination skill level, and changes in care coordination attributed to training. Finally, systems-level change was assessed to understand if increasing caregiver competence might impact the care they receive. Participants also responded to a series of questions on general demographics, type of insurance, and child’s health conditions.

**Table 2:** Measures and data collection schedule for CCEF evaluation

Measure	Number of items	Type	Pre-assessment	Post-training	1 Year follow-up
Demographics and child’s health condition(s)	8	Multiple choice	✓		✓
Training quality					
Satisfaction	4	4-point Likert scale		✓	
Content knowledge	10	Multiple choice		✓	
Suggestions to improve training tools	1	Open ended		✓	✓
Training objectives met	16	5-point Likert scale		✓	
Training impact					
Readiness for change	2	4-point Likert scale		✓	
Plans to use training information	1	Open ended		✓	
Care coordination skill level	6	4-point Likert scale	✓		✓
Use of training tools	5	Multiple choice			✓
Change attributed to training	6	4-point Likert scale			✓
System-level change					
Question from National Survey of Children with Special Health Care Needs	44	Likert scale Multiple choice	✓		✓

#### Post-training Quality Improvement

At the post-training assessment, participants responded to questions about the degree to which they were satisfied with the training as a whole and with the facilitators (responses on a four-point Likert scale from “Very Satisfied” to “Very Dissatisfied”). Participants provided written response to an open-ended question about what suggestions they had to improve the training. To assess whether the training was sufficiently teaching intended content, participants were asked to respond to ten multiple choice items around content addressed in the training. Items ask about a range of topics covered including definitions of a Medical Home, importance of transition to adult care, communicating with healthcare professionals, and navigating health insurance.

Participants’ perception as to whether training objectives were met were assessed using 16 items. The assessment used a 5-point Likert scale ranging from “Strongly Disagree” to “Strongly Agree.” Items include topics covered in the curriculum (e.g., “After today’s training I can identify the components of a Medical Home”; “After today’s training I have increased knowledge of the Patient Protection and Affordable Care Act (ACA) as it applies to CYSHCN”; and “After today’s training I understand the need to plan for child’s transition to adulthood”).

#### Training Impact

*Readiness for change* using two Likert-type items and an open ended question was assessed following the training. The 4-point Likert scale ranges from “Definitely” to “Not at all” on “Do you see the role you play in child’s healthcare changing as a result of this training?” and “How likely are you to include new individuals on your child’s care team?” Participants also provided written responses to how they might use the information from this training to improve care coordination for their child in the future.

*Care coordination skill level* was assessed at two times to compare their perception prior to training and the 1 year follow-up. The participants report skill level on their communication with doctor on 4 point Likert-type scale with responses ranging from “Very comfortable” to “Uncomfortable.” Participant responded to questions as it relates to their comfort with “Asking questions during a medical or healthcare appointment,” “Determining the best communication method to use with your child’s doctor or healthcare provider,” “Contacting your child’s doctor or other healthcare provider to get advice in between face-to-face appointments,” and “Reflecting on the appointment after it occurs to determine if you got the answers you needed.” Responses to ability to organize information (1 item) and peer support (1 item) were also a 4 point Likert-type scale with responses ranging from “Very well” to “Not at all” in response to “How well do you feel you organize information?” and “Describe how well the peer support you currently have in-place meet your needs.”

A final measure of *change attributed to the CCEF training* participation was 6-items collected in the 1 year follow-up survey. This Likert-type scale has four possible responses including “No, I didn’t change anything,” “I made a few changes,” “I made many changes,” and “Doesn’t apply; I didn’t need to make changes.” Participants responded to behavior changes they perceived as a result of the training. Questions include: “Did you make any changes to how you communicate with your child’s doctor(s) as a result of the training?” “Did the training prompt you to start a conversation with your child’s doctor(s) about transition to adult care?” “Did you change how you manage your child’s health insurance as a result of the training?” “Did you make any changes to how you organize your child’s health information as a result of the training?” “Did you make any changes to your peer supports as a result of the training?” and “Did you change how you take care of yourself as a result of the training?” The evaluation used two additional items that assessed participant’s use of 11 care coordination tools in the past year that were a part of the training curriculum.

#### System-Level Services

Question from the National Survey of Children with Special Health Care Needs (NS-CSHCN) was used to assess the child’s access to health care services including medical home, adequate health insurance, care coordination, access to needed services, transition planning and shared decision making. The NS-CSHCN questions were administered during the pre-assessment and the 1-year follow-up to the training. All MCHB outcomes were constructed using the Child and Adolescent Health Measurement Initiative’s SPSS codebook (CAHMI 2012).

## Results

### Training Quality

#### Satisfaction

Participants report a high level of satisfaction with the training with 99% reporting “satisfied” or “very satisfied” with the training and the trainers. In addition, 99% of participants “agreed” or “strongly agreed” that they had opportunities to contribute to the conversation during the training. When asked what could be improved about the CCEF training, the majority of participants explicitly said they would change nothing.

#### Content Knowledge

On average, participants answered 89.0% of items correctly on the 10 quiz-style items to assess understanding of core training content following the training (*M* = 88.9; *SD* = 13.4). A general linear model was used to understand if quiz scores varied by race, ethnicity, or income. Participants scores significantly varied by income levels (*F*(4,145) = 2.48, *p* = .046). Post-hoc comparisons of the income groups using a Bonferroni correction showed that participants in the lowest income category (< $20,000: *M* = 83.27, *SD* = 16.5) scored significantly lower than participants in the highest two income brackets ($40,001–$50,000: *M* = 92.82, *SD* = 8.98, *p* = .038; > $50,000: *M* = 92.08, *SD* = 11.79, *p* = .006). Hispanic participants scored significantly lower than other participants (Hispanic: *M* = 81.78, *SD* = 16.23; Others: *M* = 88.77, *SD* = 13.80; *F*(1,164) = 4.41, *p* = .037). No significant differences among additional racial groups and no significant interactions were found in the sample.

#### CCEF Objectives Met

Participants strongly endorsed that the 16 training objectives were met (80.9–100% on individual objectives). The only two objectives receiving an endorsement under 90% were associated with insurance (increased knowledge of the ACA as it applies to CYSHCN, and techniques for dealing with denied insurance claims). The mean of all objectives was 4.57 (*SD* = 0.38) on a five point scale with higher values indicating stronger agreement that objectives were met. Responses to objectives were compared across race, ethnicity, and income categories using a general linear model to understand if participants from different backgrounds had different perspectives on the efficacy of the training. There were no significant differences among these groups.

### Training Impact

#### Readiness for Change on Post-training Assessment

The majority of participants (88.9%) respond they were either “definitely” or “somewhat” likely to include new individuals on their child’s care team and 83.7% reported they either “definitely” or “somewhat” saw their role in their child’s healthcare changing as a result of the training. These items were not significantly correlated with the number of people currently helping them with care coordination. African American participants were more likely to endorse the most positive response option by reporting they “definitely” saw their role in their child’s healthcare changing as a result of the training (76.5% compared to 51.5% of other participants, *χ*^*2*^(3,166) = 8.54, *p* = .036). There were no other significant differences in responses to these items by race, ethnicity, or income. When asked how they will use information from the training to improve care coordination, qualitative analysis of participants’ responses reflected the following themes: organization of child’s health information (35.3%), insurance (18.8%), medical home (18.8%), resources (18.8%), advocacy (17.6%), communication (12.9%), and transition (10.6%).

#### Care Coordination Skills

Participants’ responses to items about care coordination-related skills (organization of child’s health information, peer supports, and communication with care providers) before and 1 year after the training are reported in Table 3. Participants’ reports of peer support and communication with care providers increased at statistically significant levels from the pre-training to 1 year follow-up assessments. Family income, race, ethnicity, number of health conditions, and number of people helping with care coordination were not significantly associated with the skills at follow-up.

**Table 3:** Participants’ perception of care coordination skills

Care coordination skill^a^	Care Coordination: Empowering Families (CCEF) follow-up respondents (N = 80)			
	Prior to CCEF	1 Year follow-up	*F*	*p*
	*M(SD)*	*M(SD)*		
Peer support	2.69 (0.80)	3.05 (0.74)	13.29	< .001
Communication with care providers	3.37 (0.54)	3.57 (0.56)	7.39	.008
Organization of child’s health information	3.01 (0.70)	3.14 (0.67)	2.55	.11

^a^Four point Likert scale with 4 being more positive and 1 being least positive

#### Change Attributed to CCEF

Participants reported that the training inspired them to make changes to their care coordination-related skills and activities (organization, peer support, communication with care providers, navigating insurance, and self-care), even if they already rated themselves as highly skilled in these areas (see Table 4). Participants reported using an average of three resources from the training (*M* = 3.05, *SD* = 1.98) in the past year. Local tips and resources that were collected from participants during the training and the Region 4 Midwest’s publication, *Partnering with Your Doctor: The Medical Home Approach Guide* were the most frequently referenced resources. Peer networks were also sustained after the training, with 25% of participants reporting they had remained in contact with someone from the training. When asked explicitly about transition planning, 71% of families with children 12 years or older said the training prompted them to have a conversation with their healthcare provider about transition in the past year. There were no significant differences among race, ethnic, or income groups.

**Table 4:** Participants’ attributed behavior change to CCEF training at 1 year follow-up (N = 80)

Care coordination skill	Made changes due to training (%)
Organization of child’s health information	83.8
Peer support	60.1
Communication with care providers	72.5
Navigating insurance	46.3
Self-care	65.1
Transition	71.0

### Systems-Level

The pre-assessment and 1 year follow-up surveys measured five of the six MCHB core outcomes to test whether the CCEF training would empower families to bring about change in their child’s healthcare system. At pre-assessment, there were few significant associations between demographic factors (race, income, family size, age of child with genetic condition, and number of child’s health conditions) and the likelihood that a participant would meet the MCHB outcome. Participants who did not meet MCHB outcome #5 (access to community-based services) were more likely to be White (*χ*^*2*^(1,110) = 7.51, *p* = .006).

**Fig. 3:**
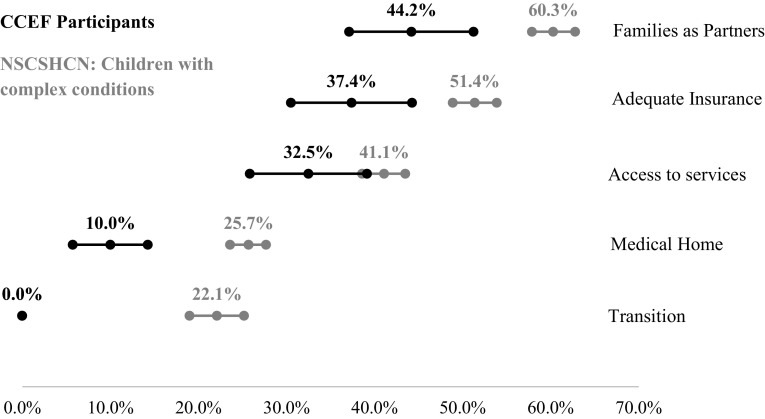
MCHB Core outcomes: comparing care coordination participants with National Survey of Children with Special Healthcare Needs respondents qualified on 4 or 5 screener criteria

We wanted to understand how the CCEF participants compared to the national sample from the NS-CSHCN. It became clear that the percentage of CCEF participants achieving the MCHB outcomes at pre-assessment was significantly lower than the national sample. Given the medical complexity of the children in the CCEF sample, we looked at national survey sub-groups that might be a better comparison. The closest sub-group we could find that mirrored “medical complexity” was the breakdown of the national sample based on the number of screener items they qualified for (1, 2, 3, and 4 or 5). When compared with the most medically complex group (those who qualified for 4 or 5 screener criteria), the CCEF sample still had significantly lower rates of meeting every MCHB core outcome (Families as Partners: *t*(3601) = 6.03, *p* < .001; Medical Home: *t*(1613) = 10.18, *p* < .001; Adequate Insurance: *t*(3002) = 4.84, *p* < .001; Access to Services: *t*(2431) = 6.93, *p* < .001; Transition: *t*(519) = 20.24, *p* < .001; see Fig. 3).

We used repeated measures general linear models to compare the percentage of participants who met the MCHB outcomes before and 1 year after the training (see Table 5). Outcome #3 (adequate insurance) reached statistical significance. The likelihood of meeting outcome #3 1 year after the training was not associated with insurance type (private, public, or both) or reports of changes to insurance over the past year (including changes due to the ACA). Outcome #1 (families are partners in decision making) was a statistical trend.

**Table 5:** Participants’ rates of meeting MCHB core outcomes at pre-assessment and 1 year follow-up

MCHB core outcome	CCEF follow-up respondents (*N* = 80)			
	Pre-assessment	1 Year follow-up	*F*	*p*
Families of CSHCN are partners in decision-making for child’s optimal health	43.8%	57.1%	3.64	.06
CSHCN receive coordinated, ongoing, comprehensive care within a medical home	6.3%	11.4%	1.34	.25
CSHCN have adequate private and public insurance to pay for the services they need	30.0%	53.8%	15.93	< .001
Community-based service systems are organized so CSHCN can use them easily	31.4%	41.7%	0.28	.60
Youth with special health care needs receive services necessary to make a successful transition to adult life (age 12 and older^a^)	0%	8.7%	2.13	.16

^a^There are 23 children aged 12 and older at pre-assessment and 28 at 1 year follow-up

Participants’ reports of the help they receive with care coordination did not significantly change a year after the training. The majority of participants (73.2%) reported that no one else helps them coordinate care for their child. Prior to the training, 6.3% of participants report one care coordinator, and 20.5% report having two or more care coordinators for their child. One year later, this picture had not changed significantly—69.5% reported that no one else helps them coordinate care, 2.4% have one care coordinator, and 26.8% had two or more care coordinators for their child. Reports of help with care coordination did not significantly vary by race, ethnicity, income, or number of child’s health conditions. Family caregivers who do have help listed between 1 and 12 individuals as people who actively help coordinate care. These individuals were most likely to be a family member or someone in a doctor’s office. Participants still averaged around one person at follow-up (*M* = 0.94, *SD* = 1.79) compared to pre-assessment (*M* = 0.81, *SD* = 1.66).

## Discussion

### Training Efficacy

The CCEF training curriculum is unique in that it targets families as the agents of change within the larger ecological context of their child’s healthcare. When families are educated about the systems they need to navigate and their role and power within them, they can bring about positive change in how they interact with the systems that support their children. Participants’ reports of increased peer support and better communication with healthcare providers in the year since the training are examples of parents as agents of change. Qualitative responses from participants suggest that education around key systems (such as insurance) and concepts (such as medical home), made them reconsider their roles as caregivers and resulted in their becoming more proactive in the coordination of their children’s care. A powerful example of parents being proactive is seen in findings around planning for transition to adult care. While the overall rate of families who met the MCHB transition outcome (based on reports of *physicians’* behavior) remained low, 71% of families with children 12 years or older (n = 28) reported initiating conversation about transition after training.

In an effort to improve access to care for underserved populations, the Region 4 Midwest staff incorporated principles of health equity during training development, recruitment, and implementation. Evaluation results demonstrated few significant differences across race, ethnic, and income groups. Where differences were found, they did not point to a consistent bias, suggesting that training efficacy was found across a broad range of participants. Of most concern is the finding that participants from the lowest income level and Hispanic participants scored significantly lower on the post-training quiz items assessing training content. To improve the quality of the training for Hispanic participants, some modifications have been made to the curriculum and evaluation material, including translating the curriculum and evaluation material for Spanish speaking participants. The Spanish version of the curriculum has not been pilot tested. Further, it should be noted that our target was a seventh grade reading level. Although reading level is sometimes confounded by inclusion of health condition names. Further study is needed to determine if some participants had trouble understanding the questions, or if this pattern of findings indicates that some participants need additional outreach and support in order to effectively apply the CCEF training curriculum to their lives.

One of the important ways families are empowered through the training is by networking with peers. The training format requires participants to interact with peers throughout a full day of facilitated activities. Like other parent-to-parent programs, the experiential learning helps connect families with each other (Hartman et al. 1992) and support each other through reciprocity of emotional and informational support (Santelli et al. 1997). These important connections help families who are raising children with complex health needs feel part of a broader community. The local connections made at the training provide another important point of contact for families. Indeed, 1 year later, 25% of participants reported being in contact with other families they met at the training. These connections may be especially important for families who have children with undiagnosed or rare conditions (around 23% of all training participants), who may not have a condition-specific support network to participate in.

The comprehensive evaluation demonstrates that four out of five participants met all training objectives. Participants have shown an increase in key knowledge and skills areas such as care coordination, medical home, transition, advocacy, importance of self-care, evaluating resources, and navigating health insurance. Besides significant differences in insurance from the pre-assessment to 1 year follow-up, a notable trend is impact on parents as partners in decision-making for the children’s health. A significant difference in communication with care providers from the pre-assessment and 1 year follow-up further supports the trend of parents as partners in decision-making. Based on participant self-reports of positive effects of the training at 1-year follow up, it is likely that improved confidence and proficiency in interacting with systems that support their children persist over time.

### Lessons Learned

Some evaluation results illustrated areas where the curriculum and additional support for participants may be warranted. For example, feedback from the training objectives indicates need to expand the ACA/insurance content. A supplemental training or linking participants to a patient navigator for individualized assessment of insurance coverage, needs and denied claims might improve access issues. Participants’ reporting the intention of adding new partners to help with care coordination immediately after the training did not align with reports of actual care coordination support 1 year later.

Use of MCHB core outcomes as measured by NS CSHCN provides an excellent comparison to understand training participants and growth over time. However, these items assess participants’ experiences with their healthcare providers, not necessarily their own experiences coordinating care for their children. Region 4 Midwest searched for other established measures that would assess the caregivers’ experience more closely. Ultimately, the evaluation team had to consider the importance of using normed and validated measures while keeping the burden on participants reasonable. Increases in meeting the core outcomes may reflect an awareness of expectations for their provider due to the training. Several participants mentioned such a change after learning about the medical home concept. We should also note that the evaluation tool has gone through several iterations since the pilot with even greater input from family participants about the evaluation design.

### Limitations

Self-report data are always liable to response biases. However, the present evaluation shows no more concern than other similar designs. There is no indication that these data were of more concern with the present evaluation than with other similar designs. Attrition in long-term follow-up is a common problem in longitudinal studies. The 1 year follow-up assessment had a 42% response rate. Additional analysis suggest that there were no significant differences in demographics or satisfaction with the training for those participants that did and did not complete the 1 year post training assessment. While the response rate is respectable for an evaluation effort one full year after participation in the training, there may be opportunity to improve the response rate by maintaining contact with participants throughout the year, requesting updated contact information, and providing incentives to complete the follow-up survey. The CCEF pilot was limited to participants from seven states within the Midwest region.

The small sample size and geographic location of the pilot limit the generalizability of the findings. Further evaluation efforts should include larger samples from other regions to see if training outcomes can be replicated in other regions of the country and with other facilitators. Another limitation is the lack a control group in the evaluation design. This project could benefit from funds to conduct a randomized control trial to determine whether care coordination skills for participants completing the training are significantly different than similar parents who do not have access to CCEF. A randomized control trial with a larger sample size will be an important next step in moving this promising practice toward an evidenced-based intervention.

## Conclusions for Practice

The CCEF training shows promise for improving care coordination-related skills among caregivers of CYSHCN from diverse backgrounds. While Hispanic participants scored lower on the English version of post-training quiz items, African American participants were more likely to endorse role in their child’s healthcare changing as a result of the training. All other evaluation findings seem to indicate similar benefit across race and ethnicity. By empowering caregivers with knowledge and skills related to care coordination, these caregivers are more able to affect positive change within their child’s healthcare systems. By partnering with HRSA funded programs and family and disease specific organizations in the recruitment of training participants, the training curriculum shows promise for use by these initiatives and others in addition to genetic services and the regional genetic networks. With a pilot expansion effort already underway, CCEF may become an evidenced-based training that certified facilitators from other HRSA grantees can use to improve core outcomes for clients they serve.
